# Metabolic, renal, and hematological changes in chronic hepatitis C patients achieving rapid virologic response after 12 weeks of direct-acting antiviral treatment: A prospective cohort study

**DOI:** 10.1371/journal.pone.0290235

**Published:** 2023-09-01

**Authors:** Phuong Nguyen Thi Thu, Dung Hoang Van, Mai Ngo Thi Quynh, Ngan Tran Thi, Khue Pham Minh, Linh Pham Van

**Affiliations:** 1 Haiphong International Hospital, Haiphong, Vietnam; 2 Hai Phong University of Medicine and Pharmacy, Hai Phong, Vietnam; Kaohsiung Medical University, TAIWAN

## Abstract

The impact of direct-acting antivirals (DAA) therapy on lipid and glucose metabolism and kidney function in patients with hepatitis C virus (HCV) infection, along with its side effects on blood cells, remains controversial. Therefore, we conducted a study that enrolled 280 patients with HCV infection who achieved sustained virologic response after treatment with DAA therapy without ribavirin to evaluate the metabolic changes, renal function, and anemia risk based on real-world data. This study was an observational prospective study with a follow-up period of 12 weeks after the initiation of DAA therapy. Data on biochemical tests, renal function, blood counts, viral load, and host genomics were recorded before treatment and after 12 weeks of treatment with DAAs. DAA therapy reduced fibrosis-4 scores and improved liver function, with significant reductions in aspartate transaminase, alanine aminotransferase, and total bilirubin levels. However, DAA therapy slightly increased uric acid, cholesterol, and low-density lipoprotein cholesterol levels. It significantly reduced fasting blood glucose levels and hemoglobin A1C index (HbA1C) in the study group, while hemoglobin (Hb) and hematocrit (HCT) concentrations decreased significantly (4.78 ± 21.79 g/L and 0.09% ± 0.11%, respectively). The estimated glomerular filtration rate (eGFR) decreased by 12.89 ± 39.04 mL/min/1.73m^2^. Most variations were not related to the genotype, except for Hb, HCT, and HbA1C. Anemia incidence increased from 23.58% before treatment to 30.72% after treatment. Patients with HCV-1 genotype had a higher rate of anemia than did patients with genotype 6 (36.23% vs. 24.62%). Multivariate analysis showed that the risk of anemia was related to female sex, cirrhosis status, fibrosis-4 score, pretreatment eGFR, and pretreatment Hb level. The results of our study can provide helpful information to clinicians for the prognosis and treatment of HCV infection.

## Introduction

Hepatitis C virus (HCV) infection is a leading cause of chronic hepatocellular damage, cirrhosis, and hepatocellular carcinoma. The goal of treatment of this disease is a sustained virologic response (SVR), which is defined as undetectable viral ribonucleic acid (RNA) at six months after the end of treatment [1]. Rapid virologic response (RVR) and complete early virological response (cEVR) were reported to be related to SVR to HCV infection treatment. Patients who fail to achieve a cEVR (defined as the absence of detectable HCV RNA after 12 weeks of treatment) or a partial early virological response (pEVR; defined as a ≥2-log_10_ decrease from pretreatment level in HCV RNA at the 12th week of treatment) have a lower likelihood of achieving SVR with an additional 36 weeks of treatment [2,3].

Interferon-containing regimens, which were first developed for the treatment of HCV infection, are correlated with low cure proportions and multiple adverse events [4]. The advent of direct-acting antivirals (DAAs) has resulted in major improvements, with cure rates for HCV infection reaching approximately 100%, along with a short mean duration of therapy and with relatively fewer side effects [5]. The early 21^st^ century saw a significant change in hepatitis C treatment guidelines with the introduction of DAAs. DAAs target key stages of the HCV life cycle, thus leading to a higher treatment response with fewer side effects than those observed with traditional therapy with interferon and ribavirin (RBV).

HCV is a single-stranded RNA virus that is classified into eight major genotype groups (HCV genotype 1 [HCV-1] to HCV genotype 8 [HCV-8]) [6,7]. There is no evidence of viral-encoded RNA-dependent RNA polymerase; therefore, these viruses evolve rapidly and the sequence diversity is extremely high, with a 31%–33% difference among genotypes and a 20%–25% difference among subtypes [8]. Although some other genotypes are also widely distributed, clear epidemiological and geographic patterns are associated with other genotypes, such as the predominance of genotype 6 in Southeast Asia and South China [9]. Different HCV genotypes have different clinical manifestations, fatty liver severity, viral loads, and variations in regulation of lipid metabolism [10,11]. Chronic infection with HCV-1 and HCV-3 genotypes differentially manifest as fatty liver and virological responses to therapies containing interferons and DAAs. Induction of lipogenic, lipolytic, and interferon-stimulated gene pathways was reportedly enriched in HCV-1 injury, whereas a broad range of immune-associated pathways is associated with HCV-3 genotype injury [12].

Variations in lipid and glucose profiles in patients with HCV infection who receive DAA therapy have been reported in several studies [13–15]. The impact of DAA therapy on lipid and glucose metabolism and on kidney function and its side effects on blood cell count remains controversial. Therefore, we conducted a prospective observational study to evaluate changes in the general metabolic processes, renal function, and blood cell count (erythrocytes and platelets) in patients with chronic HCV infection who were treated with DAA therapy and followed up for 12 weeks, as well as to determine the association between these changes and HCV genotypes.

## Materials and methods

### Study design

This study was an observational and prospective study with a follow-up period of 12 weeks after the initiation of DAA therapy. The study was conducted at Hai Phong International General Hospital from January 25, 2019, to December 30, 2022. Research data were collected from electronic medical records and via medical examination by physicians. Patients were examined, and their drug compliance and test indicators were assessed.

### Participants

Our study recruited patients from the Gastroenterology and Hepatobiliary Clinic of Hai Phong International General Hospital from January 25, 2019, to December 30, 2022, using the following inclusion criteria:

- Patients aged ≥18 years
- DAA-naive patients with HCV infection
- Patients prescribed DAAs
- Patients achieve SVR after 12 weeks of DAA treatment

Patients with the following criteria were excluded from the study:

- Patients with current hepatocellular carcinoma
- Patients with renal failure with an estimated glomerular filtration rate (eGFR) of <30 mL/min/1.73 m^2^
- Patients indicated for other regimens of HCV treatment.
- Patients prescribed drugs that are known to have drug-drug interactions with DAAs (screening by using UpToDate^®^) [16].

Chronic HCV infection was defined as the presence of detectable viral replication for at least six months [17]. DAA therapy in our study consisted of Ledvir (LDV)/sofosbuvir (SOF; Mylan Laboratories Limited; 90 mg/400 mg) and velpatasvir (VEL)/SOF (Epclusa, Gilead Sciences; 100 mg/400 mg). Medication adherence of patients was defined in terms of the amount of therapy received. Patients receiving the “full dose” of DAA therapy were considered as being adherent to treatment. Daily adherence was assessed by a daily questionnaire and by performing pill counts at each visit. HCV patients were tested and treated based on the guidelines of Study of Liver Diseases and the Infectious Diseases Society of America [18–20]. Other medications for co-morbid disease were continued.

### Data collection

For all patients, the following data were collected: demographic characteristics such as age, sex, weight, and height at baseline; characteristics of disease status; comorbidities; cirrhosis status; aspartate aminotransferase-to-platelet ratio index (APRI) score; fibrosis-4 factor (FIB-4) index; coinfection with hepatitis B virus; coinfection with human immunodeficiency virus; aspartate aminotransferase (AST); alkaline phosphatase (ALP); total bilirubin (BILT); direct bilirubin (BILD); alpha-fetoprotein; acid uric; cholesterol; high-density lipoprotein (HDL) cholesterol; low-density lipoprotein (LDL) cholesterol; triglyceride; fasting glucose; hemoglobin A1C (HbA1C); hemoglobin (Hb); hematocrit; platelet count; serum creatinine; viral load; and HCV genotype at baseline and after 12 weeks of treatment. In a dedicated section of the electronic case report form, the APRI was calculated as follows [21]:

$$APRI=\frac{AST/ULN}{Platelet\;count\;x\;100}$$

FIB-4 was calculated as follows:[22]

$$FIB-4=\frac{\left(age\;x\;AST\right)}{Platelet\;count\;x\;\sqrt{ALT}}$$

Note: *ULN = upper limit of normal range (34 U/L for female and 36 U/L for male patients)

eGFR was calculated using the following formula: [23]

$$eGFR\;(mL/min/1.73\;m^{2})=[\left(140-age)\times Wt/(0.814\times S.Cr\;in\;\mu mol/L\right)]\times (0.85\;if\;female\;patient)$$

Note: Wt: weight; S.Cr: serum creatinine level

Anemia was defined as Hb levels < 12.0 g/dL in female patients and <13.0 g/dL in male patients [24]. Mean corpuscular volume (MCV) was defined as a measure of the average volume of a red blood cell; anemia was classified into three categories depending on the patients’ MCV level: macrocytic anemia (>100 fl), normocytic anemia (80–100 fl), and microcytic anemia (<80 fl).

SVR12 was defined as cEVR achievement (undetectable HCV RNA at 12 weeks posttreatment).

HCV RNA concentration (viral load) was assessed using Roche COBAS AmpliPrep/COBAS TaqMan version 2 (Roche, Pleasanton, CA, USA) according to the manufacturer’s instructions, with a lower limit of quantification and detection of 15 IU/mL before (baseline viral load) and after 12 weeks of treatment with DAA therapy. For HCV genotyping, viral RNA from HCV RNA-positive samples was reverse transcribed using the Superscript III RT and RNaseOUT kit (Invitrogen Life Technologies, Paisley, UK), as described previously [9].

### Statistical analysis

A logistic regression model was built using R statistical software version 3.2.4 (R Core Team, Vienna, Austria) [25]. We used forward selection based on the chi-squared test of the change in residual deviance. Differences among groups were analyzed using the chi-squared test for qualitative variables, one-way analysis of variance for continuous variables with normal distributions, and the nonparametric Kruskal–Wallis test for continuous variables with nonstandard distributions. Analysis of variance was used to compare the differences in clinical laboratory indicators (such as AST, ALP, BILT, and BILD) among genotypes. The comparison between baseline and after 12 weeks of treatment with DAA was performed according to McNemar’s test. Differences were considered statistically significant if *P ≤* 0.05.

### Ethical considerations

The study protocols were reviewed and approved by the Institutional Review Board (IRB) of Hai Phong International Hospital, Vietnam (IRB.20.302). The study was conducted in accordance with the Declaration of Helsinki and International Conference on the Harmonization of the Technical Requirements for the Registration of Pharmaceuticals for Human Use—Good Clinical Practice guidelines. Prior to data collection, ethical approval was obtained from the ethics subcommittees of Hai Phong International Hospital. All patients provided written informed consent prior to the commencement of the study.

## Results

**Fig 1:**
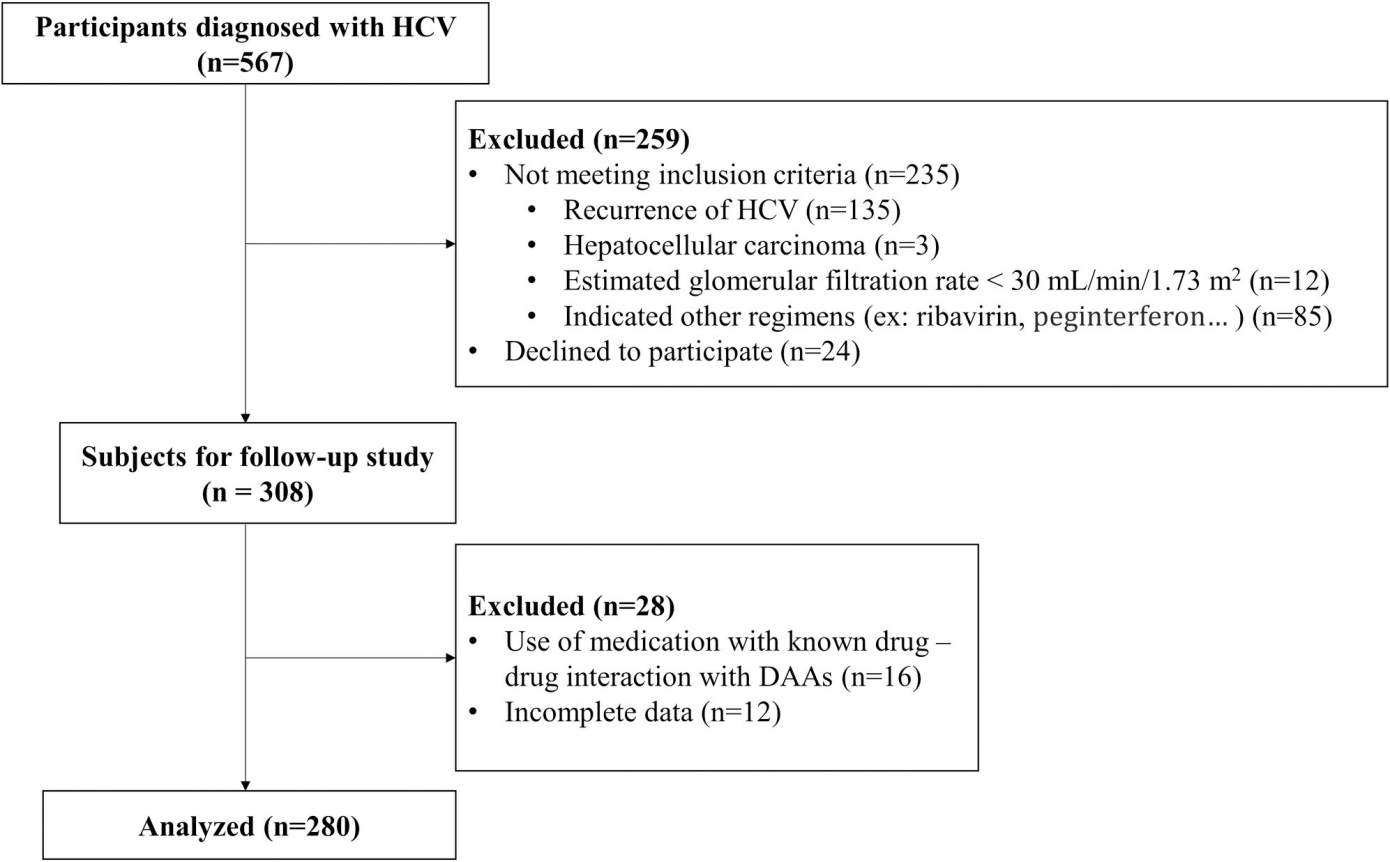
Flowchart of the study.

After screening 567 patients diagnosed with HCV infection, 280 patients who met the inclusion criteria were enrolled in our study (Fig 1).

Table 1 summarizes the clinical and demographic characteristics of the study patients. Half of the patients in our study were older than 49 years (median [IQR] = 49 [26]). Overall, 32% of the patients were male, with a mean body mass index (BMI) of 19 ± 3 kg/m^2^. The mean systolic and diastolic blood pressures before treatment were 122.86 mmHg and 73.33 mmHg, respectively. The median viral load was 4.48 log_10_ IU/mL (IQRs range 3.47–5.1 IU/mL).

**Table 1:** Clinical and demographic characteristics of the patients (n = 280).

Demographic data	Finding
Age, years, median (IQR)	49 (42,59)
Sex (male), n (%)	91 (32)
Weight, kg, mean (SD)	58.77±9.71
Height, cm, mean (SD)	161.64±7.86
BMI, mean (SD)	19±3
baseline SBP, mmHg, mean (SD)	122.86±14.8
baseline DBP, mmHg, mean (SD)	73.33±9.46
Viral load log_10_ IU/mL, median (IQR)	4.48 (3.47, 5.1)
Cirrhosis, n (%)	37 (13.16)
HCC incidence, n (%)	0 (0)
Comorbidity	
Diabetes, n (%)	25 (9)
Hypertension, n (%)	18 (6)
Ulcer, n (%)	5 (2)
HBV-HCV co-infection, n (%)	13 (5)
Malignancy (not including HCC)	6 (2)
DAA regimens	
LDV/SOF, n (%)	186 (66.43)
VEL/SOF, n (%)	94 (33.58)
Ribavirin, n (%)	0 (0)
RVR, n (%)	37 (13.22)
ARPI score, median (IQR)	0.67 (0.42, 1.33)
FIB-4 score, median (IQR)	2.09 (1.26, 3.55)
HCV genotype	
Genotype 1, n (%)	127 (45)
Genotype 2, n (%)	14 (5)
Genotype 3, n (%)	9 (3)
Genotype 6, n (%)	130 (46)

RVR: Rapid virologic response; HBV: Hepatitis B virus; HCV: Hepatitis C virus; HCC: Hepatocellular carcinoma; LDV/SOF: Ledipasvir/sofosbuvir; VEL/SOF: Velpatasvir/sofosbuvir; APRI: Aspartate aminotransferase-to-platelet ratio index; FIB-4: Fibrosis index based on four factors; BMI: Body mass index; SBP: Systolic blood pressure; DBP: Diastolic blood pressure.

Among the 280 patients with HCV infection, 25 had diabetes, 18 had hypertension, 13 had hepatitis B virus infection, 5 had gastric ulcers, and 6 had malignant cancer (excluding patients diagnosed with liver cancer). We found no HCV patients who developed HCC after 12 weeks of DAA treatment. Regarding drug regimens, 66.43% of the patients received DAA therapy with the active ingredient being LDV/SOF, whereas the remaining 33.58% received DAA therapy with the active ingredient being VEL/SOF. No patient received ribavirin in our study. Overall, RVR was achieved in 37 patients (13.22%) after 12 weeks of treatment. Among the 308 patients with HCV infection, 280 patients met the study inclusion criteria and attained SVR12 via DAA therapy. The median APRI and FIB-4 scores before treatment were 0.67 and 2.1, respectively. Regarding HCV genotypes, 46% of the patients had genotype 6, 45% had genotype 1.5%, 5% had genotype 2, and 3% had genotype 3.

**Fig 2:**
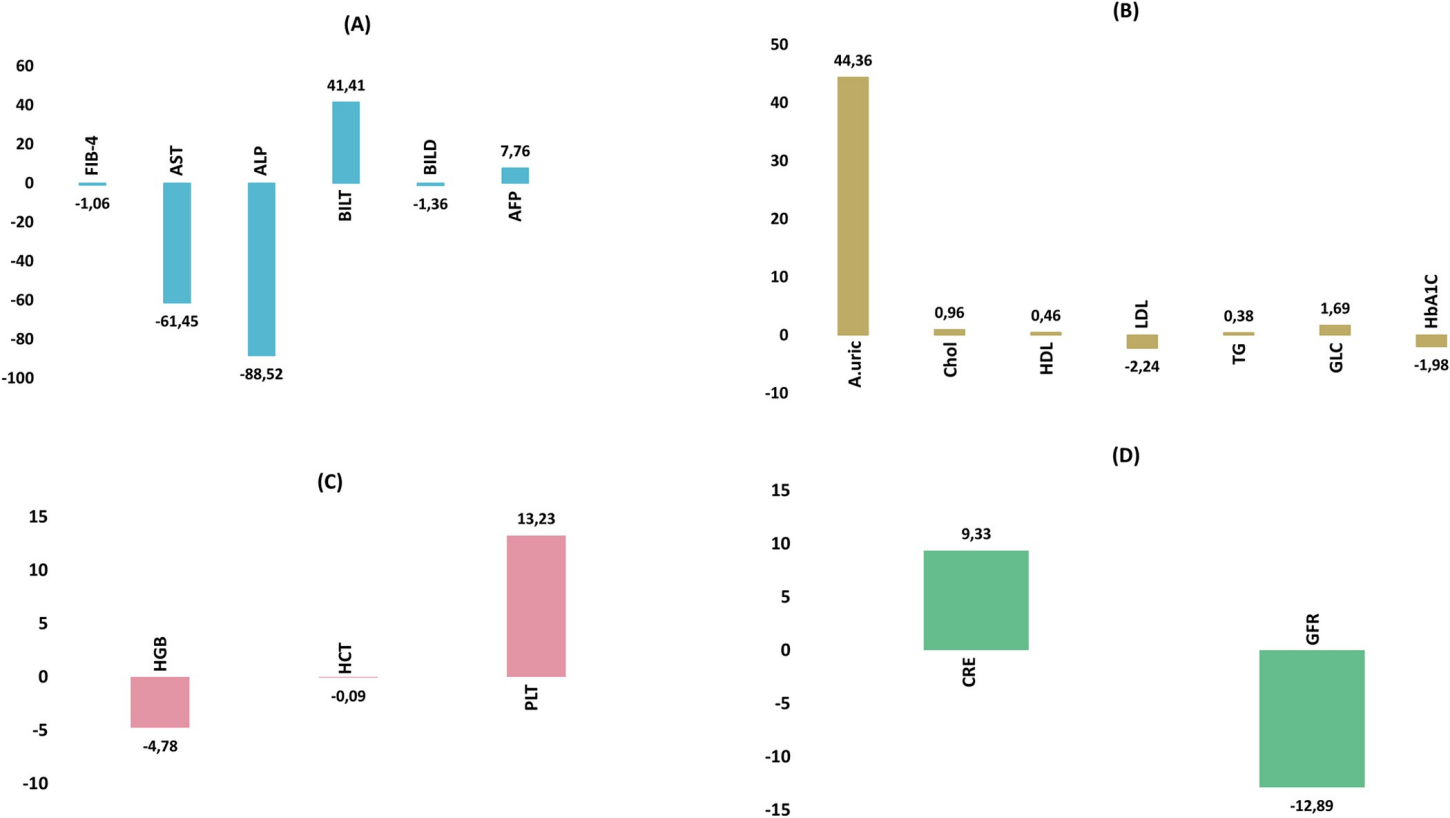
Metabolic, renal, and hematological changes after 12 weeks of direct-acting antiviral treatment (n = 280). AST: Aspartate aminotransferase; ALP: Alkaline phosphatase; HDL: High-density lipoprotein; LDL: Low-density lipoprotein; HbA1C: Hemoglobin A1C; GFR: Glomerular filtration rate; PLT: Platelet count; CRE: Creatinine; HCT: Hematocrit; BILT: Total bilirubin; A.uric: Acid uric; TG: Triglycerid; GLC: Glucose; Chol: Total cholesterol.

The comparison of hepatobiliary, renal, and metabolic functions before treatment and after 12 weeks of treatment is shown in Table 2 and Fig 2. The FIB-4 score of the study group tended to decrease by approximately 1.06 ± 2.18 after 12 weeks of treatment with DAA therapy. Liver function improved significantly after 12 weeks of treatment, as shown by the AST (IU/mL) and ALT (IU/mL) levels, which decreased sharply from a median of 50.82 and 61.73 before treatment to 28.77 and 27.25, respectively (p < 0.001), after treatment. The BILT concentration of patients in the study group reduced by 41.41 ± 84.66 mg/dL after treatment compared with the concentration before treatment (from an average of 55.17 mg/dL to 13.77 mg/dL) (p < 0.001). However, the levels of BILD and alpha-fetoprotein did not change significantly (p > 0.05). After comparing the changes in the profile of serum glucose, lipid, and uric acid metabolism between before treatment and after 12 weeks of treatment with DAA therapy, uric acid concentration increased slightly from 347.98 ± 82.66 μmol/L to 392.34 ± 144.29 μmol/L (p < 0.001). Although the total cholesterol levels of the patients increased slightly from 4.39 mmol/L to 5.35 mmol/L (p < 0.001), the LDL cholesterol concentration increased significantly from 3.17 mmol/L to 5.4 mmol/L (p < 0.001). Additionally, HDL cholesterol concentration significantly increased from 1.08 mmol/L to 1.54 mmol/L (p < 0.001). Statistical analysis showed that after 12 weeks of treatment with DAAs, triglyceride levels did not change significantly. When comparing the glucose metabolism status of patients, we found that the fasting blood glucose concentration and HbA1C index of patients with HCV infection decreased significantly (from 7.98 mmol/L to 6.29 mmol/L and from 8.14% to 6.16%, respectively) (p < 0.001). When analyzing the changes in blood counts, patients tended to have decreased Hb concentration and hematocrit index (140.99 to 136.58 g/L and from 0.42 g/L to 0.34 g/L, respectively, p < 0.05) after 12 weeks of treatment with DAAs. Similarly, platelet counts decreased slightly from 230.92 g/L to 217.7 g/L (p = 0.037). When analyzing the changes in kidney function, although the increase in creatinine concentration in the study group was not statistically significant, the clear decrease in the eGFR from 93.07 mL/min/1.73m^2^ to 80.08 mL/min/1.73m^2^ was statistically significant (p < 0.001).

**Table 2:** Data at baseline and after 12 weeks of treatment with DAA therapy (n = 280).

	Baseline (n = 280)	At 12^th^ week (n = 280)	∆*	p-value^#^
**Changes in hepatobiliary function**				
FIB-4 score, median (IQR)	2.09 (1.26,3.55)	1.26 (0.89,1.90)	-1.06±2.18	<0.001
AST (IU/L) (median (IQR))	50.82 (34.64,91.56)	28.77 (21.41,43.08)	-88.52±272.2	<0.001
ALP (IU/L) (median (IQR))	61.73 (38.16,119.81)	27.25 (16.75,57.8)	-61.45±255.98	<0.001
BILT (mg/dL) (mean ± SD)	55.17±82.51	13.77±20.22	41.41±84.66	<0.001
BILD (mg/dL) (mean ± SD)	5.71±14.68	4.36±13.47	-1.36±13.84	0.26
Alpha-fetoprotein (ng/mL) (mean ± SD)	16.08±107.16	23.83±134.47	7.76±165.48	0.452
**Changes in glucose, lipid, and uric acid metabolism**				
Uric acid (μmol/L) (mean ± SD)	347.98±82.66	392.34±144.29	44.36±174.56	<0.001
Cholesterol (mmol/L) (mean ± SD)	4.39±0.91	5.35±3.21	0.96±3.29	<0.001
HDL cholesterol (mmol/L) (mean ± SD)	1.08±0.52	1.54±1.08	0.46±1.2	<0.001
LDL cholesterol (mmol/L) (mean ± SD)	3.17±1.02	5.4±3.53	-2.24±3.49	<0.001
Triglyceride (mmol/L) (mean ± SD)	1.86±1.83	2.23±3.18	0.38±3.55	0.085
Glucose (mmol/L) (mean ± SD)	7.98±5.69	6.29±2.79	1.69±6.06	<0.001
HbA1C (%) (mean ± SD)	8.14±2.05	6.16±1.77	-1.98±2.92	<0.001
**Changes in blood count**				
Hemoglobin (g/L) (mean ± SD)	140.99±17.96	136.58±19.68	-4.78±21.79	0.004
Hematocrit (%) (mean ± SD)	0.42±0.06	0.34±0.08	-0.09±0.11	<0.001
MCV (fl) (median, min, max)	88.68 (80.27,96.92)	87.39 (80.26,96.85)		0.094
Platelet count (g/L) (mean ± SD)	217.7±67.56	230.92±81	13.23±80.81	0.037
**Changes in kidney function**				
Serum creatinine (μmol/L) (mean ± SD)	75.91±38.93	85.24±81.48	9.33±80.69	0.09
GFR (mL/min) (mean ± SD)	93.07±43.05	80.08±23.28	-12.89±39.04	<0.001

#: Student’s t-test for mean comparison and Wilcoxon rank sum test for median comparison.

*: ∆ = level at 12th week–level at baseline.

AST: Aspartate aminotransferase; ALP: Alkaline phosphatase; HDL: High-density lipoprotein; LDL: Low-density lipoprotein; HbA1C: Hemoglobin A1C; GFR: Glomerular filtration rate; MCV: Mean corpuscular volume.

Regarding differences in the clinical laboratory indicators among genotypes, changes in the hepatobiliary function of the patients were not related to the HCV genotypes (p > 0.05). Similarly, the changes in lipid profiles after 12 weeks of treatment with DAAs (cholesterol, HDL cholesterol, LDL cholesterol, and triglycerides) did not differ among the genotypes (p > 0.05). Regarding changes in blood glucose profiles and HbA1C index, although the analysis of blood glucose levels among genotypes did not show a statistically significant difference (p = 0.302), the change in HbA1C index after 12 weeks of treatment showed a statistically significant difference among the various HCV genotypes (p = 0.041) (Table 3). Hemoglobin and hematocrit levels after 12 weeks of treatment with DAAs in all HCV genotypes were reduced when compared with pre-treatment data (Table 3). Additionally, these reductions were significantly different between genotypes (p<0.05).

**Table 3:** Relationship between changes in clinical laboratory r and hepatitis C viral genotypes (n = 280).

Index	HCV-1	HCV-2	HCV-3	HCV-6	p (ANOVA)	r^2^
ΔALP (IU/L, mean ± SD)	-77.5±232	-39.7±75.9	28.4±25.5	-108.8±326.6	0.303	0.004
ΔAST (IU/L, mean ± SD)	-70.7±285.4	-25±55.2	-16.9±14.5	-59.5±249	0.803	0.001
ΔBILT (mg/dL, mean ± SD)	-41.6±87	-54.5±97.9	-65.7±96.8	-38.2±81	0.662	0.001
ΔUric acid (μmol/L, mean ± SD)	43±181.8	38.1±173.5	40.6±101.1	46.8±173.8	0.847	0.001
ΔChol (mmol/L, mean ± SD)	1.2±3.3	2±5.8	-0.3±1.8	0.8±3.2	0.283	0.005
ΔHDL cholesterol (mmol/L, mean ± SD)	0.5±1.3	0.4±0.4	1.1±2.2	0.5±1.1	0.416	0.011
ΔLDL cholesterol (mmol/L, mean ± SD)	-2.1±3.5	-1.2±3.3	-4±3.6	-2.5±3.6	0.244	0.015
ΔGlucose (mmol/L, mean ± SD)	-2±7.2	-3.6±7.3	-0.1±2.1	-1.4±4.9	0.302	0.004
ΔHbA1C (%, mean ± SD)	-2.6±2.9	-0.8±3.9	-2±3.6	-1.6±2.8	0.041	0.01
ΔHGB (g/L, mean ± SD)	-8±21.2	-0.5±23.9	-15.9±20	-1.5±21.9	0.031	0.032
ΔHCT (%, mean ± SD)	-0.11±0.11	-0.04±0.12	-0.08±0.13	-0.07±0.11	0.041	0.03
ΔPLT (g/L, mean ± SD)	-14.6±88.2	-32±69.7	-22.3±131.9	-9.3±70.4	0.754	0.005
ΔCRE (g/L, mean ± SD)	4.4±22.3	8.9±14.2	4.7±25.9	14.6±116.4	0.315	0.004
ΔGFR (mL/min, mean ± SD)	-8.6±34.4	-10.9±20.1	-11.6±44.6	-17±44.1	0.344	0.011

∆ = level after 12 weeks of treatment–level at baseline; AST: Aspartate aminotransferase; ALP: Alkaline phosphatase; HDL: High-density lipoprotein; LDL: Low-density lipoprotein; HbA1C: Hemoglobin A1C; GFR: Glomerular filtration rate; PLT: Platelet count; CRE: Creatinine; HCT: Hematocrit; BILT: Total bilirubin; Chol: Total cholesterol.

Table 4 shows the comparison of anemia incidence before and after treatment in the 280 patients and according to HCV genotypes. Upon comparing the anemia status of patients newly diagnosed with HCV infection, we observed that among the 280 patients, the number of those with anemia increased from 66 (23.58%) before treatment to 86 (30.72%) after treatment (p = 0.047). Before treatment, the incidence of anemia in patients with HCV-1 infection was not different from that of patients with HCV-2 infection and HCV-6 infection (p > 0.05), but this incidence was lower than that of patients with HCV-3 (20.48% vs. 33.34%, p = 0.03). After 12 weeks of treatment with DAAs, the incidence of anemia in patients with HCV-1 infection was only slightly different from that of patients with HCV-6 infection (36.23% vs. 24.62%, p = 0.04). In the 14 patients with HCV-2, HCV-3, and HCV-6, the incidence of anemia before and after treatment and among genotypes was not significantly different (p > 0.05).

**Table 4:** Comparison of anemia incidence before and after 12 weeks of treatment with DAAs in the study population and among HCV genotypes (n = 280).

	Baseline		p-value*	After the 12^th^ week		p-value*	p-value^#^
	anemia (n [%])	non-anemia (n [%])		anemia (n [%])	non-anemia (n [%])		
Total (n = 280)	66 (23.58)	214 (76.43)		86 (30.72)	194 (69.29)		0.047
HCV-1 (n = 127)	26 (20.48)	101 (79.53)	0.09 (HCV-2) 0.03 (HCV-3) 0.54 (HCV-6)	46 (36.23)	81 (63.78)	0.56 (HCV-2) 0.63 (HCV-3) 0.04 (HCV-6)	0.004
HCV-2 (n = 14)	6 (42.86)	8 (57.15)	1 (HCV-3)	4 (28.58)	10 (71.43)	0.43 (HCV-3)	0.683
HCV-3 (n = 9)	3 (33.34)	6 (66.67)	0.69 (HCV-6)	4 (44.45)	5 (55.56)	0.24 (HCV-6)	1
HCV-6 (n = 130)	31 (23.85)	99 (76.16)	0.19 (HCV-2)	32 (24.62)	98 (75.39)	0.75 (HCV-2)	1

*: Comparison among HCV genotypes at baseline or after 12 weeks of treatment with DAAs, Fisher’s exact test

^#^: Comparison between baseline and after 12 weeks of treatment with DAA, according to McNemar’s test.

Multivariate logistic regression analysis showed that the anemia status of patients with HCV infection after week 12 of treatment was dependent on sex (odds ratio [OR] with 95% confidence interval [CI] = 5.87 [2.87, 12.83], p = 0.001), cirrhosis status (OR [95% CI]: 2.21 [1.98, 5], p = 0.047); FIB-4 (OR [95% CI]: 1.18 [1.02, 1.4], p = 0.046), baseline eGFR (OR [95% CI]: 0.99 [0.99, 1], p = 0.004), and baseline Hb concentration (OR [95% CI]: 0.97 [0.95, 0.99], p = 0.001) before treatment (Table 5).

**Table 5:** Multiple logistic regression analysis showing the factors associated with anemia in patients with HCV infection after 12 weeks of DAA therapy (n = 280).

Coefficients	Estimate	Odds ratio (95% CI)	p-value
Sex (female)	1.77	5.87 (2.87, 12.83)	0.001
Cirrhosis (yes/no)	0.8	2.21 (1.98, 5)	0.036
FIB-4	0.16	1.18 (1.02, 1.4)	0.046
Baseline viral load (log_10_ IU/mL)	0.11	1.11 (0.89, 1.41)	0.379
Baseline eGFR (mL/min)	-0.02	0.99 (0.99, 1)	0.004
Baseline Hb (g/L)	-0.04	0.97 (0.95, 0.99)	0.001

CI: Confidence interval; FIB-4: Fibrosis index based on four factors; eGFR: Glomerular filtration rate; Hb: Hemoglobin.

## Discussion

We evaluated the changes in metabolic processes, renal function, and blood counts of patients with chronic HCV infection who were treated with DAA therapy and followed up for 12 weeks. Our prospective study of 280 patients with HCV infection treated with DAAs revealed changes in metabolism and blood counts after treatment when compared with findings before treatment. Our study showed that HCV-1 infection accounted for approximately 45% of the 280 patients with chronic HCV infection. This rate is quite similar to the results of a previous multicenter study of 282 patients, which showed that 60% of patients with HCV were infected with HCV-1 [27]. In addition, this study showed that HCV-6 (73.9%) was quite common in the group of patients requiring dialysis and multi-transfusion; however, HCV-6 was noted in 46% of patients in the current study. We also found two rare genotypes in Vietnamese individuals: HCV-2, which infected 14/280 patients, and HCV-3, which infected 9/280 patients. In total, 1.8% (n   =  5) and 0.4% (n  =   1) of patients with HCV-6 and HCV-3, respectively, were detected among the 282 Vietnamese patients of the previous study [27].

FIB-4 index is a simple noninvasive test that is based on routine biochemical results, including platelet count, AST, ALT, and age. It can be used to assess the degree of liver fibrosis and has been shown to be consistent with the FibroTest [11]. The median index (IQR) of the FIB-4 score of the 280 patients in our study was 2.09 (1.26, 3.55). A previous multicenter retrospective study of 6,632 patients with sustained virological chronic HCV infection showed a mean FIB-4 index of 2.66 ± 1.98 before treatment [28]. Similarly, a study of 392 patients who received DAA therapy for chronic hepatitis C infection revealed that the mean FIB-4 score decreased significantly from 2.54 (1.65, 4.43) to 1.80 (1.23, 2.84) (p < 0.001) [29]. Our study also showed a significant decrease in FIB-4 scores from 2.09 (1.26, 3.55) to 1.26 (0.89, 1.90).

Our study showed a decrease in AST, ALT, and BILT levels after treatment compared with the levels before treatment. Our research results also agree with those of studies by other authors. In a retrospective study of 115 patients with HCV infection who achieved SVR12, 85.5% and 83.9% of patients showed normalization of ALT and AST levels, respectively. After 12 weeks of treatment, the percentage of the normalization of ALT and AST levels increased to 90.8% [30].

A previous cross-sectional observational study of 97 patients with HCV infection who were treated with DAAs showed that after 12 weeks of DAA therapy, the gamma-glutamyl transferase, AST, and ALT levels decreased (p < 0.001) [15]. Both ALT and AST are biochemical indicators of liver damage, including HCV-mediated hepatocellular injury. Our research shows that DAA therapy not only clears HCV RNA but also improves liver function and restores liver cells. Our study showed that the serum uric acid concentration of patients increased after treatment compared with the concentration before treatment (p < 0.001). In a previous study, 15.8% of the 373 patients with chronic hepatitis receiving antiviral therapy had hyperuricemia [31]. In a previous study, logistic regression analysis revealed that factors associated with hyperuricemia in male patients included BMI and advanced fibrosis, whereas factors associated with hyperuricemia in female patients included eGFR and diabetes mellitus. Furthermore, hyperuricemia was observed in 7.5% of patients with chronic hepatitis C infection, and this was associated with LDL cholesterol level, arterial hypertension, eGFR, and severity of steatosis, according to multivariate logistic regression analysis [32].

Our study showed an increase in total cholesterol levels of approximately 0.96 mmol/L after 12 weeks of treatment with DAAs. LDL cholesterol and HDL cholesterol levels increased from 3.17 ± 1.02 mmol/L to 5.4 ± 3.53 mmol/L and from 1.08 ± 0.52 mmol/L to 1.54 ± 1.08 (p < 0.001), whereas triglycerides levels remained unchanged. A systematic review and meta-analysis of 14 studies (n = 1,537 patients) indicated an increase in total cholesterol levels at 12 weeks after initiation of treatment (+15.86 mg/dL; p < 0.001) [14]. The LDL trend was similar to the change in total cholesterol levels in the overall analysis. In a previous study, a mean increase in the HDL cholesterol level of 3.36 mg/dL (95% CI: 0.92, 5.79; p = 0.07) was observed after 12 weeks of treatment. Furthermore, DAAs induced mild lipid changes in patients with chronic hepatitis C infection who were treated with DAAs [14]. Furthermore, our study showed that fasting serum glucose concentration and HbA1C index decreased significantly after 12 weeks of treatment with DAAs. In contrast to our study findings, a prospective study of 105 patients who achieved SVR and were treated with DAAs demonstrated no change in blood glucose levels [33]. A previous prospective observational cohort study of 128 patients with HCV infection and type 2 diabetes showed that both fasting blood glucose levels and HbA1C index decreased after 12 weeks of treatment with a minimal reduction of 0.5% in the HbA1C index [34].

Our study showed a decline in renal function at the early stage of treatment with DAAs, with a reduction in eGFR from 93.07 ± 43.05 mL/min/1.73m^2^ to 80.08 ± 23.28 mL/min/1.73m^2^ (p < 0.001). Similar to our study, a cohort study of 1,536 patients with chronic HCV infection treated with DAAs also showed a decrease in eGFR. Furthermore, a previous study performed multivariate analysis for renal function decline from baseline to SVR24 and showed that liver transplantation, hypertension, and baseline eGFR of <60 mL/min/1.73 m^2^ were independent risk factors [35]. However, O’Donnell A et al analyzed data from 1,390 electronic health records of DAA-naive patients and found that DAAs may not be nephrotoxic; furthermore, in the short-term, HCV clearance may not improve CKD [36]. In a study by Huang CF et al., they examined the effects of sofosbuvir-based and non-sofosbuvir-based regimens on 12,995 patients with chronic hepatitis C (CHC) from the Taiwan nationwide real-world HCV Registry Program. Among the participants, 6,802 were treated with sofosbuvir-based regimens, while 6,193 received non-sofosbuvir-based regimens. They found that both sofosbuvir and non-sofosbuvir-based regimens restored renal function in CHC patients with CKD, especially in those with significant renal impairment. Multivariate adjusted analysis demonstrated that baseline eGFR >90 mL/min/1.73 m^2^ was the only factor that was independently associated with significant slope coefficient differences of eGFR (−1.98 mL/min/1.73 m^2^; 95% confidence interval, −2.24 to −1.72; P < .001). Notably, the use of sofosbuvir was not independently associated with changes in eGFR [37]. In contrast, another study evaluated the renal function of 96 diabetic and 187 non-diabetic patients after the completion of DAA treatment. The results showed that DAAs improved renal function in diabetic patients with HCV who had an eGFR < 60 mL/min/1.73 m^2^ or were ≥ 65 years of age, irrespective of the antiviral regimen administered [38].

Anemia is a well-documented occurrence in patients with HCV infection receiving pegylated interferon and RBV regimens [39–41]. A previous retrospective study involving 152 patients receiving RBV + DAA revealed that the incidence of anemia was 15.1%. Multivariable logistic regression analysis showed that eGFR, baseline Hb level, duration of 12 weeks of treatment, and decrease in Hb% (weeks 0–2) were significantly associated with the likelihood of developing anemia (p < 0.05) [42]. Additionally, the relative incidences of anemia for specific DAAs were 10% for sofosbuvir/ledipasvir and between 7.5% and 10.5% for ombitasvir/paritaprevir/ritonavir plus dasabuvir, depending on the duration of treatment (12 or 24 weeks), respectively [38,43].The incidence of anemia in our study population was quite high because it increased from 23.58% before treatment to 30.72% after treatment (p = 0.047). Anemia is one of the most common problems encountered in clinical practice. The causes of normocytic anemia with an MCV of 80–100 fL include acute bleeding, iron deficiency, anemia of chronic disease/inflammation, bone marrow suppression (cancer, aplastic anemia, infection), chronic renal insufficiency, hypothyroidism, hypopituitarism, malabsorption/malnutrition, excess alcohol, and copper deficiency/zinc poisoning. Some of these factors, such as malabsorption/malnutrition and excessive alcohol consumption, were not evaluated in our study. Several studies have demonstrated iron deficiency, which is one of the causes of anemia in patients with chronic HCV infection [44,45]. Our multivariable regression analysis revealed that factors such as sex, cirrhosis status, FIB-4 index, Hb level, and eGFR before treatment were related to the incidence of anemia due to HCV infection. Furthermore, compared to their male counterparts, women with HCV infection were 5.87 times more likely to develop anemia (p = 0.001). The prevalence of anemia was 2.21 times higher among patients with HCV infection who were diagnosed with cirrhosis (OR: 2.21; 97.5 CI: [1.98.5], p = 0.036). A multicenter study that enrolled 446 Japanese HCV genotype 2 patients (303 treatment-naïve and 143 treatment-experienced patients), including 190 (42.6%) patients aged ≥ 65 years and 90 (20.2%) patients with compensated cirrhosis, indicated that 10.5% of patients developed anemia with hemoglobin levels < 10 g/dL; being ≥65 years old and ITPA CC genotype were associated with the development of anemia. Because of anemia, 13.2% of the patients in this study required a reduction in the dose of ribavirin (23.2% aged ≥ 65 and 5.9% age < 65 years). Nevertheless, reducing the dose of ribavirin and the mean dose of ribavirin were not related to treatment failure, probably because almost all patients underwent 12 weeks of treatment [43,46].

Furthermore, for every one-point increase in the FIB-4 score before treatment with DAAs, the risk of anemia increases by 1.18 fold (OR: 1.18; 97.5% CI: [1.02, 1.4], p = 0.046). In contrast, each 1 mL/min/1.73m^2^ increase in eGFR before treatment reduced the risk of anemia by 1% during the 12 months of treatment with DAAs (OR [97.5% CI]: 0.99 [0.99.1]). Similarly, every 1 g/L increase in Hb level at baseline reduced the risk of anemia by 3% (OR [97.5% CI]: 0.97 [0.95, 0.99]).

Our study included a specific set of outpatients and thus may not apply to inpatients who demonstrate different characteristics. Anemia is extremely common in HCV patients who are old and often present with multiple diseases. However, our study did not consider the possible factors contributing to anemia in HCV patients such as malnutrition, alcohol consumption, PWID, and duration of hospitalization. Polymorphisms located near the gene encoding the interferon-lambda beta subunit can identify patients with a high probability of achieving SVR [47]. However, this factor mainly predicts treatment efficacy in patients receiving ribavirin. Therefore, in our study, these polymorphisms were not considered, as patients receiving ribavirin were not recruited. Another limitation of our study is that patients were not stratified depending on their combined current treatment for diseases such as diabetes, dyslipidemia, and gout, which may impact the study results. However, in the evaluation, we individually compared biochemical tests, renal function, blood counts, viral load, and host genomics before and after 12 weeks of treatment with DAAs.

## Conclusion

Our prospective study, which enrolled 280 HCV patients who achieved SVR12 after treatment with DAA therapy without ribavirin, found that DAAs improved liver function as indicated by the FIB-4 index, and AST, ALT, and BILT levels. Additionally, the fasting blood glucose level, HbA1C, and renal function of patients with HCV infection decreased significantly posttreatment. We observed that the prevalence of anemia increased from 20.48% before treatment to 36.23% after treatment (p = 0.004). Furthermore, multivariate analysis showed that the risk of anemia was related to sex, cirrhosis status, fibrosis-4 score, pretreatment eGFR, and pretreatment Hb levels. The results of our study can help clinicians guide the treatment and prognosis of this disease and improve the effectiveness and safety of therapies for chronic HCV infection.

## Supporting information

S1 File Data of the study. (DOC)

S2 File (XLSX)

## Abbreviations

BMI: Body mass index
CI: Confidence interval
LDL: Low-density lipoprotein
OR: Odds ratio
RVR: Rapid virologic response
SVR: Sustained virologic response
APRI: Aminotransferase-to-platelet ratio index
FIB-4: fibrosis-4 factor index
AST: Alanine aminotransferase
DAA: Direct-acting antiviral
HCV: Hepatitis C virus
